# Assessment of antibody assay methods in determination of prevalence of infectious bursal disease among local chickens and guinea fowls in Kwara state, North Central Nigeria

**DOI:** 10.14202/vetworld.2018.1183-1187

**Published:** 2018-08-28

**Authors:** Oluwafemi Babatunde Daodu, Oladapo Oyedeji Oludairo, Julius Olaniyi Aiyedun, Hauwa Motunrayo Ambali, Rafiu Adebisi Kadir, Oluwakemi Christiana Daodu, Isaac Dayo Olorunshola, Arimie Deborah Adah

**Affiliations:** 1 Department of Veterinary Microbiology, Faculty of Veterinary Medicine, University of Ilorin, Nigeria, Sub Sahara Africa; 2 Department of Veterinary Public Health and Preventive Medicine, Faculty of Veterinary Medicine, University of Ilorin, Nigeria, Sub Sahara Africa; 3 Department of Veterinary Medicine, Faculty of Veterinary Medicine, University of Ilorin, Nigeria, Sub Sahara Africa; 4 Department of Wildlife and Ecotourism, Faculty of Agriculture, University of Ibadan, Nigeria, Sub Sahara Africa

**Keywords:** agar gel immunodiffusion test, assessment, enzyme-linked immunosorbent assay, indirect hemagglutination test, infectious bursal disease, Kwara state, prevalence

## Abstract

**Aim:**

This study aimed to assess available assay methods for infectious bursal disease (IBD) diagnosis and seromonitoring in local birds. It also sought to know the prevalence of IBD antibodies among local chickens and guinea fowls in Kwara state, North Central Nigeria.

**Materials and Methods:**

Sera were obtained from local chickens and guinea fowls and IBD virus (IBDV) antibodies were assayed using enzyme-linked immunosorbent assay (ELISA), indirect hemagglutination (IHA) test, and agar gel immunodiffusion (AGID) test.

**Results:**

A total of 265 sera were obtained from local birds during dry and wet seasons. ELISA recorded the highest prevalence of 81.1% (215/265) while IHA and AGID detected IBDV antibodies in 183 (69.1%) and 122 (46%) birds, respectively. Significant differences were established for IBD-positive sera based on the assay method used, bird species, and seasons.

**Conclusion:**

This study indicated that ELISA is the most sensitive and reliable assay method while AGID is the least. It also showed that there is a high prevalence of IBDV antibodies among local birds which were not vaccinated, and this implies a high IBDV activity among these bird species in the study area. This may have significant epidemiological implications on the spread of the virus to exotic bird reared in the rural areas on a commercial scale. Thus, this study suggests continuous surveillance, awareness campaign, and advocacy for vaccination of indigenous birds against IBD.

## Introduction

Infectious bursal disease (Gumboro, IBD) was first observed in the area of Gumboro, in Delaware, USA [1]. The virus belongs to the family Birnaviridae, genus *Avibirnavirus*. It possesses two molecules of linear double-stranded RNA of approximately 6 kbp size [2]. It is a hardy virus and can survive under harsh environmental condition or treatment [3]. Gumboro virus is extremely contagious and causes a self-limiting disease in both domestic birds (chickens and turkeys) and wild birds (guinea fowl, quail, ducks, and pheasants) [4].

The contribution of village-reared poultry to meat production in Nigeria cannot be overemphasized. Chickens are the most important poultry species reared [5]. Apart from non-infectious diseases limiting poultry production, Gumboro disease is classified as the first infectious diseases affecting them [1]. Although commercial vaccines are available for prevention against IBD virus (IBDV) and some other infectious viral diseases, domesticated birds in villages in Nigeria are rarely vaccinated [6-8]. This might be based on overwhelming factors such as ignorance of vaccination, cost, availability of veterinarians, or licensed vaccinators to mention a few.

Several diagnostic techniques have been used in the detection of IBDV antigen, antibodies, and conserved genes. Serological assays which have been in use for diagnosis and/or confirmation of Gumboro disease include agar gel immunodiffusion test (AGID), indirect hemagglutination (IHA) test, passive hemagglutination test, enzyme-linked immunosorbent assay (ELISA), immunohistopathology test, immunoperoxidase test, counterimmunoelectrophoresis test, and immunofluorescent test. These have variable sensitivity and specificity [8-15].

Majority of the owners of these village birds are low-income earners who cannot afford the running cost of some of these techniques. Despite the severity and economic loss associated with Gumboro disease, there has not been any report of the disease in Kwara state, especially among local birds. To this end, this study aimed to detect IBDV antibody using three available serodiagnostic assays which are rapid, cheap, and accessible to the local bird keepers and to compare the sensitivity of the diagnostic assays. It also aimed to determine the prevalence of IBDV antibodies in local birds in Kwara State.

## Materials and Methods

### Ethical approval

All applicable international, national, and/or institutional guidelines for the care and use of animals were duly followed.

### Study area and sample collection

The study area was Oja-titun poultry abattoir (market) located in Ilorin metropolis, North Central Nigeria. It is a major abattoir that receives the highest number of local birds in Kwara state for sale and/or slaughter. Birds usually originate from villages within the state and neighboring states.

Immediately after slaughter, blood samples were collected from chickens and guinea fowls into sterile plain bottles and were transported to the laboratory under a cold chain. The blood was then centrifuged at 2500 rpm for 10 min to harvest the serum into a sterile Cryovial tube. Separated sera were stored at −20°C until the time of use for assay. Sampling was seasonally based and other variables such as bird species and category were recorded.

### Assay methods

Each of the sera was differently assayed using AGID test, IHA test, and ELISA. The results were entered into a spreadsheet for analyses.

### IBD antigen preparation

The bursa of Fabricius of IBDV-infected chickens was harvested and processed for virus isolation. The isolate from processed tissue was identified as IBDV using IBDV-specific hyperimmune serum [8]. The sera collected from birds and prepared viral antigen were put to use in AGID and IHA assays.

### AGID test

Immunodiffusion plates were prepared by dissolving 8 g sodium chloride in 100 ml of distilled water followed by the addition of 1.25 g agar noble. This mixture was gently mixed and boiled in a water bath until the agar is completely dissolved. The agar was allowed to cool to about 50°C before it was poured in 6 of 9 cm immunodiffusion plates and allowed to solidify. The plates were then kept overnight at 4°C before used. Using a template and well cutter (4 mm), seven wells of 4 mm (a group of six wells surrounding a center well) were made. The surrounding wells were filled with test sera while the center well was filled with IBDV antigen, and this was recorded in the template sheet. Positive and negative controls (PCX and NCX) were also included to validate the result using known IBDV-positive antigen, positive serum, and negative serum. The plates were incubated in a humid chamber for up to 35 h and read using a diffused light and recorded as positive or negative based on the presence or absence of white precipitation line, respectively, between serum and antigen wells.

### IHA test

#### Erythrocytes sensitization

Human blood group O was collected into a sterile anticoagulant tube using a sterile hypodermic syringe under sterile condition. The blood was then centrifuged at 3000 rpm for 10 min and the supernatant with white blood cell layer removed and discarded. The red blood cells were then washed 3 times using phosphate buffer saline (PBS) with the removal of supernatant at each wash.

Subsequently, one volume each of washed red blood cell (RBC) and prepared IBDV antigen was mixed, and two volume of PBS was the added. The mixture was gently shaken and incubated at 37°C for 45 min. To ensure adequate antigen-RBC contact, the mixture was shaken at intervals during incubation. Unattached antigens were later removed from the mixture by centrifugation at 3000 rpm for 10 min after which the supernatant was discarded. To ensure total removal of unbounded antigen, washing was repeated using PBS, thereby leaving behind sensitized RBC (IBDV antigen-human RBC O blood group complex).

Slide agglutination test was used to confirm the sensitization of human erythrocytes.

#### Hemagglutination test

The test sera were heat inactivated in a water bath at 56°C for 30 min while 1% sensitized erythrocytes were prepared. The IHA test was carried out as described by Hussain *et al*. [9]. Briefly, 50 µl of PBS was dispensed into all the wells of “U”-shaped bottom microtiter plate. Then, 50 µl of test serum was then dispensed in the first well of the row followed by 2-fold dilution, and subsequently, 50 µl of the mixture from the 12^th^ well was discarded. After this, 50 µl of 1% sensitized RBC was added to all the wells. PCX and NCXs were included in the test. The microtiter plate was then tapped to ensure adequate surface antibody and sensitized RBC contact. The plate was incubated at 37°C for 30 min. Sera samples showing characteristic tent formation/reticulum settling of erythrocytes at the bottom of the microtiter plate were regarded as positive while those with central button-shaped settling of erythrocytes were regarded as negative. The IHA titer results were recorded.

### ELISA

The presence of IBDV antibody was assayed using ELISA kit (IDEXX^®^ Laboratory, Inc., United States). The manufacturer’s instructions were adhered to strictly. Briefly, the protocol involved 1:500 dilution of sera sample using deionized water. 100 µl each of undiluted negative and positive controls was dispensed in duplicate wells of an antigen-coated microtiter plate. Then, 100 µl of diluted sera sample was dispensed into appropriate well. The plate was left to incubate for 30 min at room temperature (18-26°C). The solution in the wells was then removed, and each well was washed with approximately 350 µl of deionized water 3-5 times and then tapped on a paper towel to remove any residual wash fluid. This was followed by the addition of 100 µl of conjugate into each well. The plate was allowed for incubation for 30 minutes at room temperature. Removal and washing of the wells were repeated as described previously, then 100 µl of TMB substrate was dispensed into each well, and the plate was left to incubate at room temperature for 15 min. Finally, 100 µl of stop solution was added to each of the wells. The absorbance value was measured at 650 nm using ELISA reader. The result was validated based on the manufacturer’s recommendation that mean OD value of NCX must be ≤0.150, and when it is subtracted from mean OD of PCX, the result must be >0.075.

The endpoint titer of the samples was calculated using the formula Log_10_ Titer=1.09 (Log_10_ S/P)+C.

Where, S/P (sample to positive ratio)=(Sample mean−NCX)/(PCX−NCX) and C is 3.36 (relates S/P at a 1:500 dilution to an endpoint titer). The presence of IBD antibody was reported as positive when S/P ratio is >0.2 and negative when S/P ratio is ≤0.2. Furthermore, the antilogarithm of Log10 titer was calculated (IDEXX^®^ software) and recorded as the quantity of IBD antibody in each sample.

### Statistical analysis

Data were entered into GraphPad Prism version 5.03 (GraphPad Software Inc., USA) to test for significant differences in the assay methods used for detection of IBDV antibody. The statistical significance of bird species, seasons, and age group was also determined on nominal (positive or negative) and titer results using Fisher’s exact or Chi-square test and Student’s t-test (unpaired) or one-way ANOVA, respectively. Confidence interval at 95% (CI 95%) and odds ratio (OR) was also calculated. Statistical significance was assumed at p <0.05.

## Results

The number of IBDV-positive sera varied depending on assay used. ELISA detected IBDV antibody in 215 (81.1%) sera while IHA and AGID detected 183 (69.1%) and 122 (46%) IBD-positive sera, respectively (Table-1). The result also showed a significant difference in the sensitivity of various tests (AGID, IHA, and ELISA) in the detection of IBDV antibody with p<0.0001 (χ^2^=74.46; df=8). IHA titer ranged from 1:2 to 1:256 with geometric mean titer and modal titer of 3.1 and 1:16, respectively (Table-2).

**Table-1:** Comparison of AGPT, IHT, and ELISA results for detection of infectious bursal disease in local chickens and guinea fowls.

Diagnostic test	Number of sera sample	Positive sera (%)	Negative sera (%)	p-value
AGID	265	122 (46.0)	143 (54.0)	<0.0001 (χ^2^=74.46; df=8)
IHA	265	183 (69.1)	82 (30.9)	
ELISA	265	215 (81.1)	50 (18.9)	

AGID=Agar gel immunodiffusion test, IHA=Indirect hemagglutination test, ELISA=Enzymelinked immunosorbent assay

**Table-2:** IHA titer for infectious bursal disease in local chickens and guinea fowls.

Positive sera								
IHA titer	1:2	1:4	1:8	1:16	1:32	1:64	1:128	1:256
Number of samples	20	30	44	32	30	15	3	9
GMT	3.1							

IHA=Indirect hemagglutination

Due to the high sensitivity and specificity of ELISA compared with the other two tests, more insight was obtained for important variables obtained during sample collection. Among the birds, 85.8% (211/246) of chicken and 21.1% (4/19) of guinea fowl had IBDV antibody (Table-3). During dry season, 86.5% (147/170) positive sera were obtained which was higher compared to wet season samples (Table-3). Furthermore, there was a significant difference in the exposure of chickens and guinea fowls to IBDV as determined by antibody detection (p<0.0001; OR=0.04423; CI 95%=0.01387-0.1411) (Table-3). However, the difference in their IBDV antibody titers remained non-significant (p=0.2729 CI 95%=−567.8-2019) (Table 4). Significant difference was recorded for seasonal mean IBDV antibody titer (p=0.043; CI 95%=−815.0-−15.71) (Table-4) and seasonal exposure (p=0.005; OR=0.3941; CI 95%=0.2107-0.7371) (Table-3). This result indicated that birds were more exposed 0.3941 times during dry season compared to the wet season. There was no significant difference in the presence of IBDV antibody and its mean titers with regard to bird category (hen, cock, and grower).

**Table-3:** Distribution and significance of IBDV antibodypositive birds using ELISA.

Features	Number of sera	Number of positive sera (%)	*p-value	*OR (*CI 95%)
Bird species				
Chicken	246	211 (85.8)	<0.0001	0.04423 (0.01387-0.1411)
Guinea fowl	19	4 (21.1)		
Season				
Dry	170	147 (86.5)	0.005	0.3941 (0.2107-0.7371)
Wet	95	68 (71.6)		
Bird categories				
Hen	39	29 (74.4)	0.2062 (c^2^=3.158; df=2)	
Cock	21	15 (71.4)		
Grower	205	171 (83.4)		

* Inferential statistic based on positive and negative result. OR=Odds ratio, CI 95%=Confidence interval 95%, ELISA=Enzymelinked immunosorbent assay, IBDV=Infectious bursal disease virus

**Table-4:** IBDV antibody titer distribution in local chickens and Guinea fowls using ELISA.

Features	Number of positive sera (%)	^a^Mean±SEM	^b^p-value (CI)
Bird species			
Chicken	211 (85.8)	2232±94.58	0.2729 (−567.8-2019)
Guinea fowl	4 (21.1)	1507±487.5	
Season			
Dry	147 (86.5)	2097±111.4	0.0430 (−815.0-15.71)
Wet	68 (71.6)	2512±166.0	
Bird categories			
Hen	29 (74.4)	2486±245.8	0.2855
Cock	15 (71.4)	1830±303.4	
Grower	171 (83.4)	2206±106.5	

a Antilog10 titer.

b Significance calculated using the sample titers. CI 95%=Confidence interval 95%, ELISA=Enzymelinked immunosorbent assay, IBDV=Infectious bursal disease virus, SEM=Standard error of the mean

## Discussion

The study showed that ELISA recorded the highest prevalence of 81.1% (215/265) for IBDV antibody while IHA and AGID detected IBDV antibody in 183 (69.1%) and 122 (46%) sera samples, respectively. This indicated that local birds in the study area were exposed to the wild-type IBDV since they were not vaccinated. Furthermore, various techniques used indicated that ELISA is most sensitive, followed by IHA while AGID was the least sensitive assay for IBDV antibody detection. This high IBDV antibody prevalence in Ilorin, North Central Nigeria, recorded in this study is in agreement with Okwor *et al*. [8], Adene *et al*. [16], Lawal *et al*. [17], and Sule *et al*. [18], who reported the prevalence of 88.4%, 68.0%, 63.5%, and 63%, respectively, in South-eastern, North-eastern, and South-western region of Nigeria. The slight variations in IBDV antibody prevalence in various regions of Nigeria as reported by various researchers also suggest that environmental factors (especially weather and vegetation) might influence the spread of the virus [19]. In addition, differences in the prevalence of IBDV antibody observed using ELISA, IHA, and AGID in this study may be the reason for variation in prevalence rates in different regions in Nigeria since researchers used different assays. In general, ELISA has been described to be the most sensitive and specific antibody assay, followed by IHA. The least sensitive is AGID [8]. In comparison to AGID and ELISA, IHA become more useful when it is necessary to determine the antibody titer (though serum titration can be done to estimate the antibody titer). The study showed that there was a significant difference in the sensitivity among the three IBDV antibody detection techniques used (p<0.0001, χ^2^=74.46; df=8). Using ELISA, the prevalence of IBDV antibody in local chicken was 85.8% (222/246) compared to guinea fowl 21.1% (4/19). This was statistically significant (p<0.0001; OR=0.04423; CI 95%=0.01387-0.1411). This suggests that chickens might be at more risk and susceptible to wild-type IBDV, leading to a higher seroconversion rate than shown in guinea fowl.

The study further showed a significant difference in seasonal prevalence of IBDV antibody with 86.5% and 71.6% in dry and wet season, respectively. This indicated that local birds were more exposed during dry season, a period when there is intense scavenging of scarce food in the environment. This has also been observed for some viral diseases in extensively managed poultry [19]. The difference in the prevalence due to season was found to be significant (p=0.005; OR=0.3941; CI 95%=0.2107-0.7371), and it indicated that birds were more exposed 0.3941 times during dry season compared to wet season. This result is in discordant with Lawal *et al*. [17] who reported a higher prevalence in wet season than in dry season. This difference might be as a result of climatic and vegetation differences which has been reported to influence disease transmission in poultry [19].

## Conclusion

This study was able to show that of the three different accessible and cheap serodiagnostic assays available to the local bird keepers, ELISA is the most sensitive and reliable while AGID is the least sensitive. It also showed that there is a high prevalence of IBDV antibodies among local birds in the study area. There were significant differences found based on seasons and bird species. The high prevalence recorded in this study indicated a high IBDV activity among the bird species, and this may have significant epidemiological implications on the spread of the virus to exotic birds reared in the rural areas on a commercial scale. Continuous surveillance, awareness campaign, and advocacy for vaccination should be encouraged, especially among the local bird keepers.

## Authors’ Contributions

OBD and OCD conceived, designed, and collected sample for the research study. OBD, OOO, JOA and HMA, RAK, and ADA carried out the laboratory assays. OBD, OCD, IDO, and ADA analyzed data and drafted manuscript. All authors read, revised, and approved the final manuscript.
